# Editorial: Exploration of the human brain using magnetic resonance imaging and spectroscopy with transcranial direct current stimulation

**DOI:** 10.3389/fnins.2024.1538414

**Published:** 2024-12-17

**Authors:** Chang-Hoon Choi, N. Jon Shah, Ferdinand Binkofski

**Affiliations:** ^1^Institute of Neuroscience and Medicine - 4, Forschungszentrum Jülich, Jülich, Germany; ^2^Institute of Neuroscience and Medicine - 11, Forschungszentrum Jülich, Jülich, Germany; ^3^JARA - BRAIN - Translational Medicine, Aachen, Germany; ^4^Department of Neurology, RWTH Aachen University, Aachen, Germany; ^5^Division of Clinical Cognitive Sciences, Department of Neurology, RWTH Aachen University Hospital, Aachen, Germany

**Keywords:** tDCS, MRI, MRS, brain, magnetic resonance spectroscopy, transcranial direct current stimulation

The enormous progress made in brain stimulation and neuroimaging approaches in recent decades has dramatically supported the exploration and understanding of the human brain. These approaches have cast light on various brain functions and diseases and also have provided clinically and therapeutically relevant information.

Transcranial electrical stimulation (tES) encompasses a range of non-invasive brain stimulation tools, including transcranial direct current stimulation (tDCS) and transcranial alternating current stimulation (tACS). Furthermore, transcranial magnetic stimulation (TMS) is a widely used method that employs magnetic fields to induce electrical currents in the brain, modulating neuronal activity. These brain stimulation techniques influence brain function and facilitate research into neural processes and potential therapeutic applications by applying weak electrical or magnetic currents to the scalp. tDCS, a specific type of tES, has gained prominence since its introduction (Priori et al., 1998; Nitsche and Paulus, 2000), and is the primary focus of this Research Topic. This technique modulates neuronal excitability by inducing hyper- or hypo-polarization of membranes and altering energy levels in the brain. The acute effects of tDCS are observed during stimulation, while the after-effects can be monitored long after the stimulation has ceased (Bikson et al., 2019; Patel et al., 2019). The impact of tDCS on the brain is influenced by various parameters, including polarity, duration, current intensity and target area, with the choice of montages being particularly crucial (Choi et al., 2021).

Complementing tES, magnetic resonance imaging (MRI) and spectroscopy (MRS) are well-established techniques extensively utilized in clinical practice and neuroscience research. These methods allow for the *in vivo* examination of the human brain, providing excellent soft tissue contrast and detailed metabolic and functional information. MRS is particularly valuable for quantifying the concentrations of various brain metabolites, such as γ-aminobutyric acid (GABA), glutamine, glutamate, and high-energy phosphates, with high sensitivity. Positron emission tomography (PET) further supplements these imaging and spectroscopic techniques by enabling the study of metabolic processes and neuroreceptor activity.

This Research Topic includes valuable studies focusing on cutting-edge methods or advancements in using novel tDCS in conjunction with/without imaging and spectroscopic tools for the human brain. Given the potential these modalities have for illuminating various brain structures and functions, Research Topic will certainly be of great interest in the neuroscience community.

The study led by Binkofski (Patel et al.) investigated changes in GABA levels and energy metabolites in the primary motor cortex (M1) following anodal tDCS using proton (^1^H) and phosphorus (^31^P) MRS techniques. The results demonstrated the feasibility of measuring both ^1^H and ^31^P components in a single measurement, appeared to show an increase in GABA concentration, ATP/Pi and PCR/Pi ratios after stimulation.

Cohen et al. used tDCS and tACS with functional MRI (fMRI) to investigate genetic generalized epilepsy in M1, focusing on sensorimotor network alterations. Their initial findings indicated no dependency on stimulation polarity, suggesting further research with larger cohorts to understand current distribution and brain structures. Sun et al. examined refractory epilepsy patients using tES, resting-state and event-related fMRI, revealing a reduction in the small-world property of brain networks and a shift toward random configurations. This structural change could impair neural stability and cognitive functions, highlighting the complex interplay between network architecture and neurological disorders.

Kern et al. investigated the somatotopic organization and functional role of the supplementary motor area (SMA) using repetitive navigated TMS and MRI. Their study refined and validated a protocol for precise SMA mapping, including the non-dominant hemisphere and lower extremities, contributing to understanding the role of SMA in motor language function.

Sato et al.'s study assessed the effects of tDCS current direction on motor performance and cortical excitability, targeting M1 and SMA regions relevant to leg function. They found that precise electrode positioning enhanced motor performance, with A-P tDCS improving sit-to-stand repetitions and P-A tDCS increasing knee flexor strength and reducing intracortical inhibition.

Chang et al. explored auditory-motor integration in vocal pitch regulation using tDCS targeting the left dorsolateral prefrontal cortex (DLPFC). Anodal tDCS reduced peak magnitudes and prolonged peak times of vocal adjustments to pitch perturbations compared to sham stimulation, supporting the idea that the left DLPFC exerts inhibitory control over vocal feedback mechanisms through a top-down process.

Shaikh et al. used TMS, ^18^F-desmethoxyfallypride PET and MRI to study the DLPFC. They found that repeated intermittent theta burst stimulation enhanced regional prefrontal excitation and modulated the fronto-striatal network in a dose-dependent manner. Their approach monitored changes in radioligand binding, offering insights into cortical control over dopamine release mechanisms and striatal mapping.

Honda et al. examined the correlation between music rhythm processing and glutamatergic levels in the caudate using ^1^H MRS. They discovered that higher neurometabolite levels were associated with improved rhythm and meter production abilities, suggesting the importance of measuring these levels to understand the neurochemical mechanisms underlying musical rhythm processing.

Meinzer et al. reviewed the effects of tDCS on neural mechanisms underlying human cognition, addressing gaps in understanding its impact on cognitive functions in health and disease. They discussed factors contributing to variability in tDCS studies, design considerations for tDCS-fMRI research, and emphasized rigorous experimental control. The review also explored how tDCS effects vary across the lifespan and proposed establishing large-scale, multidisciplinary consortia to enhance tDCS research.
